# *Nymphanthus belliflorus* (Phyllanthaceae), a new species from Thailand

**DOI:** 10.7717/peerj.20559

**Published:** 2026-01-21

**Authors:** Piya Sukkharom, Pranom Chantaranothai, Pimwadee Pornpongrungrueng

**Affiliations:** 1 Applied Taxonomic Research Center (ATRC) and Centre of Excellence on Biodiversity (BDC), Department of Biology, Faculty of Science, Khon Kaen University, Khon Kaen, Thailand; 2 Honorary Research Associate, Royal Botanic Gardens, Kew, Richmond, Surrey, United Kingdom

**Keywords:** *Eriococcus*, Morphology, Phyllantheae, *Phyllanthus*, Taxonomy, Section *Nymphanthus*

## Abstract

*Nymphanthus belliflorus*, a newly described species from the northeastern part of Thailand, is the most similar to *N. chantaranothaii*, *N. glaucescens* and *N. huamotensis* in having staminate flowers with four sepals bearing long-fimbriate margins and the pistillate flowers composed of 5–6 sepals with long-fimbriate margins. However, it is distinguished by its swollen stem base, young branchlets that are glabrous, disc glands of staminate flowers that are obdeltoid and yellow-reddish in color, long pedicel of pistillate flowers (3.2–4.8 cm long) and fruits (3.5–5.5 cm long). The description, distribution, ecological information and provisional conservation status are provided.

## Introduction

*Nymphanthus* Lour. (Phyllanthaceae) was first described in Flora Cochinchinensis (de Loureiro, 1790) and later treated as a section within *Phyllanthus* L. (Müller, 1866). However, morphological and molecular evidence has shown the wide phenotypic variability and paraphyly of *Phyllanthus* (Hoffmann, Kathriarachchi & Wurdack, 2006; Kathriarachchi et al., 2006; Pruesapan et al., 2012; van Welzen et al., 2014; Bouman et al., 2018). In response, Bouman et al. (2021, 2022) proposed a revised classification of tribe Phyllantheae, resurrecting *Nymphanthus* as a distinct genus supported by both molecular and morphological data.

*Nymphanthus* comprises 86 species that are distributed in mainland Asia, Malesia and Australia. The genus can be characterized by four sepals, four free disc glands alternate with sepals, two or four stamens in a staminate flowers and 5–6 sepals, shallowly cupuliform to urceolate or five to rarely six free disc glands, three pistils with bifid or entire stigma in a pistillate flowers (Bouman et al., 2022). Moreover, another distinct characteristic is pollen morphology, which is spheroidal to ellipsoidal shape, pantoporate and reticulate ornamentation type with granules in the lumen (Webster & Carpenter, 2008; Chen et al., 2009). Bouman et al. (2022) divided the genus into section *Nymphanthus* characterized by its pedicellate flowers, 3-locular ovaries, and bifid stigmas, and section *Scepasma* (Blume) R.W.Bouman that has sessile or subsessile flowers, (4–) 5–8-locular ovaries, and usually entire stigmas.

In Thailand, eight species of *Nymphanthus* were reported in Flora of Thailand (previously treated under *Phyllanthus*) (Chantaranothai, 2007) and two additional species were reported in the country under *Phyllanthus* as *P. chantaranothaii* Pornp., J.Parn. & Hodk. and *P. huamotensis* Pornp., Chantar. & J.Parn. (Pornpongrungrueng et al., 2019). However, with the formal resurrection of *Nymphanthus* as a distinct genus, these two species were transferred to this genus as *N. chantaranothaii* (Pornp., J.Parn. & Hodk.) R.W.Bouman and *N. huamotensis* (Pornp., Chantar. & J.Parn.) R.W.Bouman, respectively (Bouman et al., 2022).

Recently, an uncertain *Nymphanthus* species was found in the ecotone between mixed deciduous and dry dipterocarp forest on sandstone mountains in Phu Mai Ruak (Mueang district, Sakon Nakhon province) and Phu Wiang National Park (Wiang Kao district, Khon Kaen province) in the northeastern part of Thailand. This plant is most similar to *N. chantaranothaii*, *N. glaucescens* (Miq.) R.W.Bouman and *N. huamotensis*. The taxonomic status of this plant needs to be investigated. Therefore, this study aims to investigate and compare the gross morphology of this unknown *Nymphanthus* taxon and other similar species to clarify taxonomic status of this newly discovered taxon.

## Materials and Methods

The plant samples were collected from Phu Mai Ruak, Sakon Nakhon province in the northeast of Thailand. The morphological characteristics were studied using a stereo microscope. The measurements were taken from dried specimens and compared to the herbarium specimens of similar species kept in various herbaria such as Bangkok Herbarium (BK), Bangkok Forest Herbarium (BKF), Khon Kaen University Herbarium (KKU) and Queen Sirikit Botanic Garden Herbarium (QBG) (herbarium acronym according to Thiers, 2025). This study followed the standard procedures for plant taxonomic research as outlined by Henderson (2005). Moreover, taxonomic literature of the plant from Thailand (Chantaranothai, 2007; Kantachot & Chantaranothai, 2013; Pornpongrungrueng et al., 2017; Pornpongrungrueng et al., 2019) and neighboring countries; China (Li & Gilbert, 2008; Yao et al., 2022), India (Balakrishnan & Chakrabarty, 2007; Chakrabarty & Balakrishnan, 2018), Java (Backer & Bakhuizen van den Brink, 1963), Malaysia (Ridley, 1924; Whitmore, 1973), Myanmar (Kress et al., 2003) and Vietnam (Nguyen, 2007) were investigated.

For seed morphology, mature fruits were air-dried and kept in silica gel. The seed morphological characters were studied following the methods described by Salmaki et al. (2011). Seed outlines were studied and photographed under a Nikon SMZ25 stereo microscope, and the seed coat ornamentation was investigated under LEO 1450VP scanning electron microscope (SEM). Terminology of seed morphology was based on Bojnanský & Fargašová (2007). Due to the lack of seed samples for *N. glaucescens*, thus the fruit and seed characters for this species were taken from Chantaranothai (2007).

The provisional conservation status was assessed following IUCN Red List categories and criteria (IUCN Standards and Petitions Committee, 2022).

The electronic version of this article in Portable Document Format (PDF) will represent a published work according to the International Code of Nomenclature for algae, fungi, and plants (ICN), and hence the new names contained in the electronic version are effectively published under that Code from the electronic edition alone. In addition, new names contained in this work which have been issued with identifiers by IPNI will eventually be made available to the Global Names Index. The IPNI LSIDs can be resolved and the associated information viewed through any standard web browser by appending the LSID contained in this publication to the prefix “http://ipni.org/”. The online version of this work is archived and available from the following digital repositories: PeerJ, PubMed Central SCIE, and CLOCKSS.

## Results

### Taxonomic treatment

**Figure 1:**
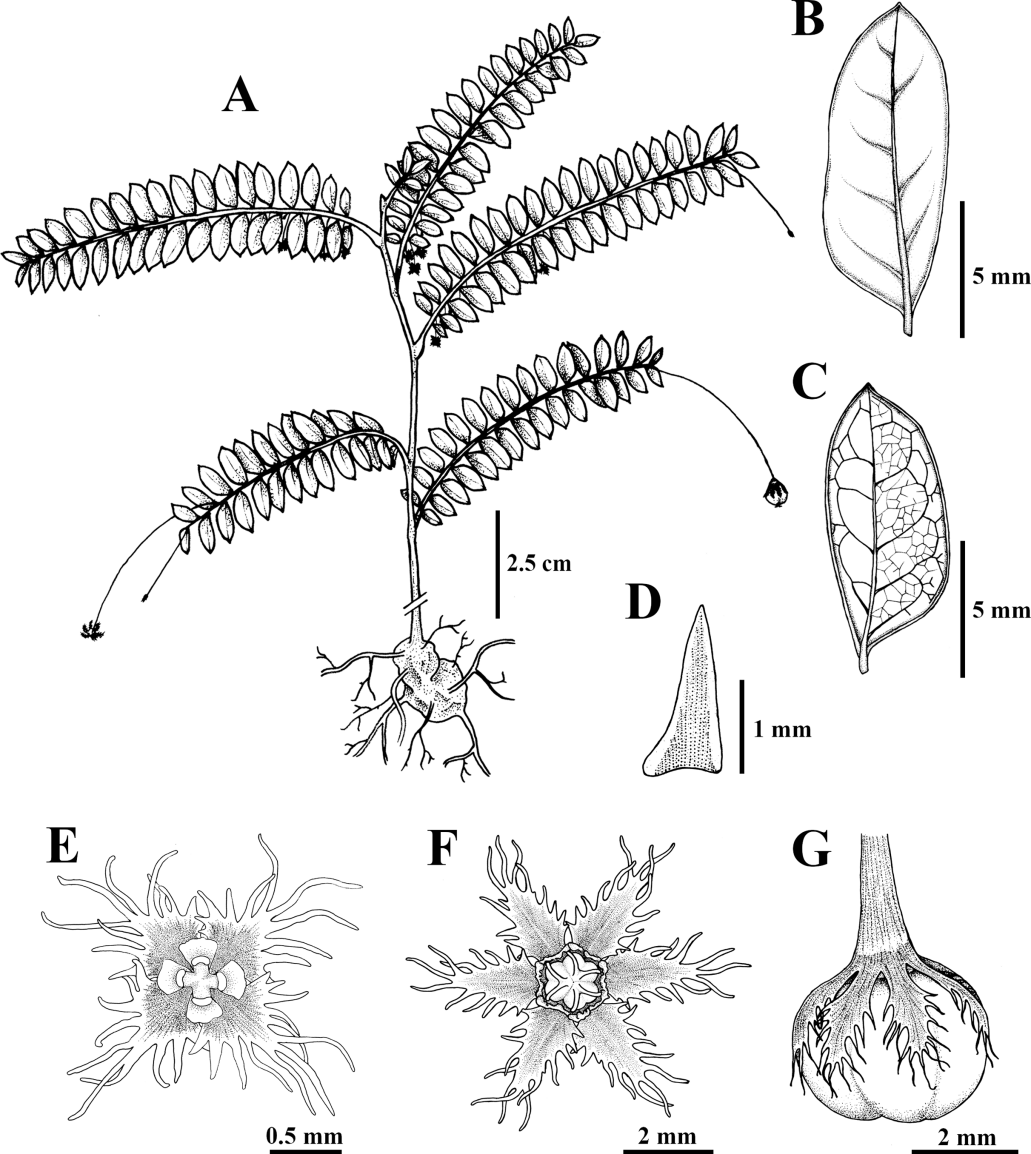
*Nymphanthus belliflorus* P. Sukkharom, Chantar. & Pornp., *sp. nov*. (A) Habit. (B) Leaf (adaxial side). (C) Leaf (abaxial side). (D) Stipule. (E) Staminate flower. (F) Pistillate flower. (G) Mature capsule. Figure drawn by Piya Sukkharom.

**Figure 2:**
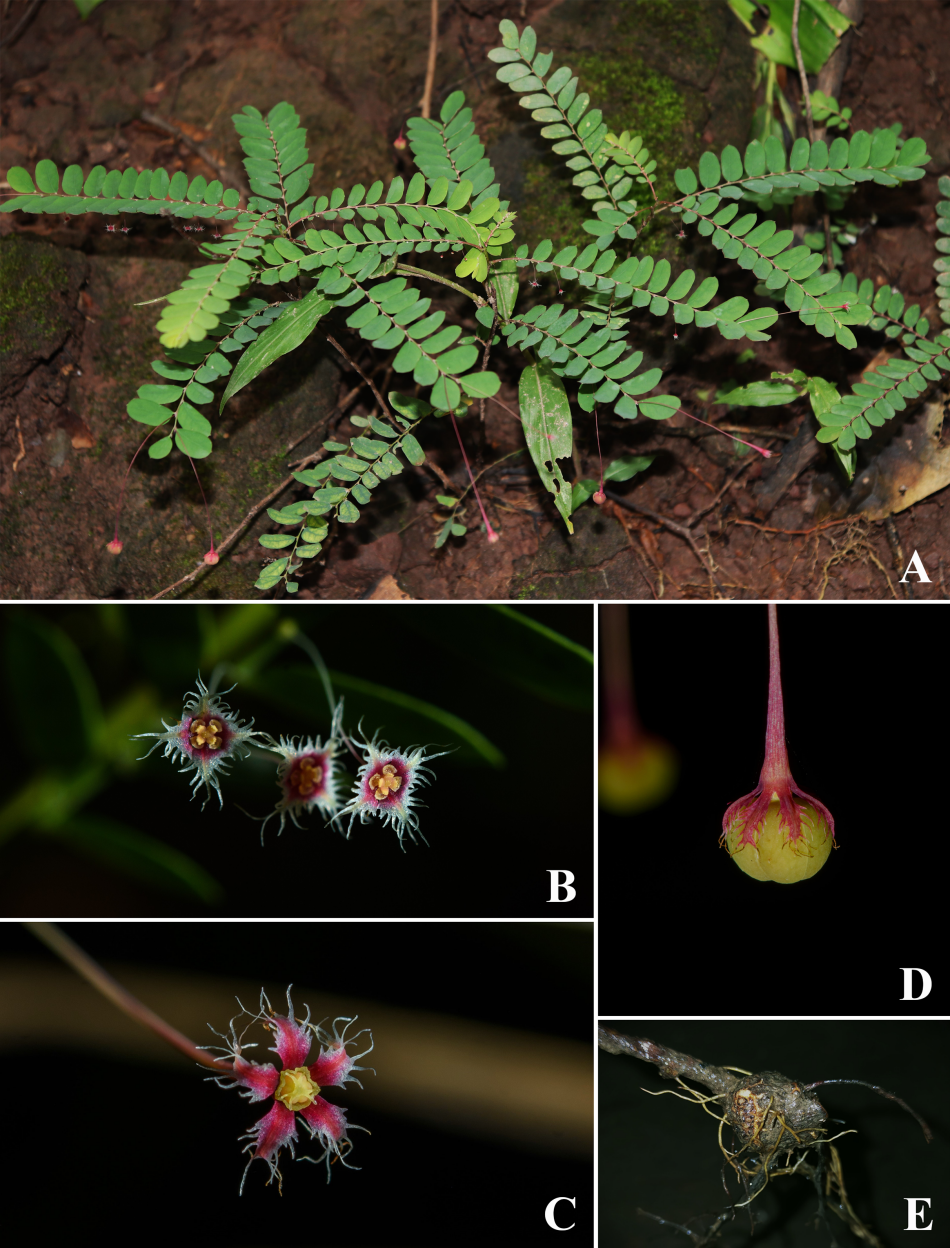
*Nymphanthus belliflorus*. (A) Habit. (B) Staminate flowers. (C) Pistillate flower. (D) Mature capsule. (E) Swollen stem base. (A) & (E) photos by Piya Sukkharom, (B–D) photos by Silakan Khunnok.

**Figure 3:**
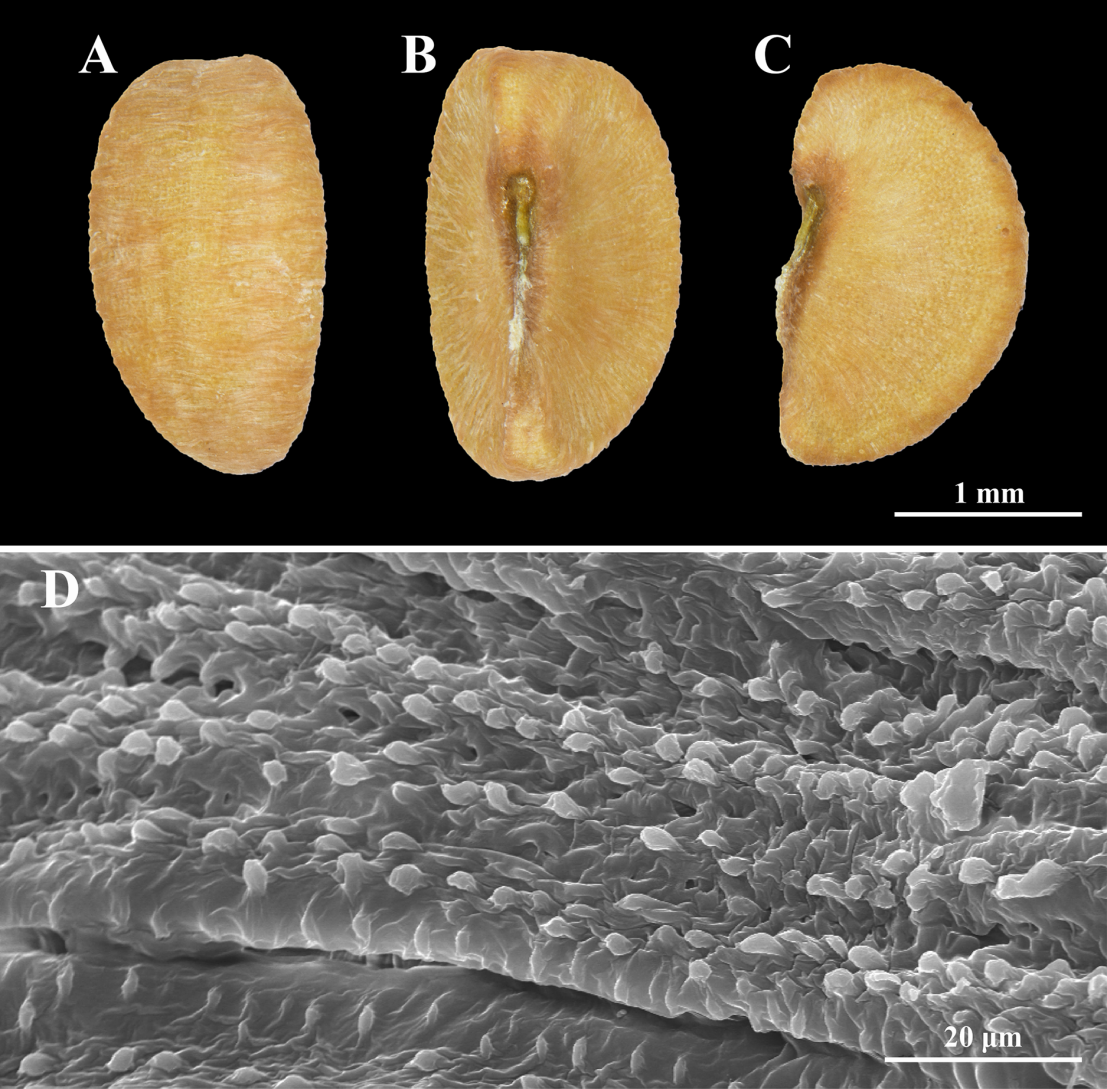
Seed morphology of *Nymphanthus belliflorus*. (A) Dorsal view. (B) Ventral view. (C) Lateral view. (D) Transversely granular striate ornamentation.

*Nymphanthus belliflorus* P. Sukkharom, Chantar. & Pornp., *sp. nov*. (Figs. 1–3)

**Type.** THAILAND. Sakon Nakhon, Mueang District, Huai Yang Subdistrict, Phu Mai Ruak, 17°5.175′N, 104°1.708′E; alt. 242 m; 30 September 2023, *P. Sukkharom & P. Pornpongrungrueng* 64 (holotype KKU!; isotypes BKF!, QBG!).

**Diagnosis.** *Nymphanthus belliflorus* is distinguished from other species of *Nymphanthus* by a combination of swollen stem base, long-fimbriate sepal margins in both staminate and pistillate flowers, obdeltoid, yellow to reddish disc glands in staminate flowers, and long pedicels in pistillate flowers and fruits (3.2–4.8 cm long and 3.5–5.5 cm long, respectively).

**Description.** Undershrubs, 20–60 cm high; branchlets terete, greenish or reddish, glabrous; stem base swollen. ***Stipules*** lanceolate-subulate, 0.6–2 by 0.2–0.8 mm, glabrous. ***Leaves*** alternate; petiole 0.2–0.5 mm long, glabrous to papillose; laminar ovate, obovate to oblong, 0.2–1.7 by 0.2–0.8 cm, membranous, base oblique, margin entire, marginate, apex mucronate or acute; lateral veins in 2–5 pairs. ***Flowers*** unisexual; staminate flowers 1–3 in axillary fascicles along the lower part of the branchlets; pistillate flowers solitary in distal axils. ***Bracts*** subulate, 0.5–1 by 0.1–0.2 mm, glabrous. ***Staminate flowers***: pedicel 6–8 mm long, reddish, glabrous; sepals 4, red with white margin, rhombic-ovate, 1.3–1.6 by ca. 1 mm, margin long fimbriate; disc gland 4, yellow-reddish at the lower part, free, obdeltoid; column ca. 0.1 mm long; anthers 0.1–0.2 mm long, transversely dehiscent. ***Pistillate flowers***: pedicel (2–)3.2–4.8 cm long, reddish, glabrous; sepals (5–)6, red with white margin, narrowly lanceolate, 2–2.8 by ca. 1 mm, margin long fimbriate; disc gland annular, 6-lobed, yellowish, surface undulate or tuberculate; ovary superior, ca. 1 mm diam., 3-locular, ovule 2 per locule, glabrous to slightly undulate; styles 3, free, ca. 0.1 mm long; stigmas almost entirely bifid, ca. 0.2 mm, glabrous. ***Fruits*** capsular, smooth, 3.3–4.8 mm diam., greenish or reddish; pedicel 3.5–5.5 cm long. ***Seeds*** trigonous, pale brown, 2.3–2.5 by 1.3–1.4 mm, surface transversely granular striate.

**Etymology.** The specific epithet refers to the beautiful flowers of this species.

**Vernacular name.** Wan thorani san lek (proposed here).

**Distribution.** Khon Kaen and Sakon Nakhon Provinces in the northeast of Thailand.

**Ecology.** *Nymphanthus belliflorus* grows on sandy soil with organic matter found in the ecotone between mixed deciduous and dry dipterocarp forest, at 240–285 m elevation.

**Phenology.** Flowering and fruiting in June–October.

**Provisional conservation assessment**. *Nymphanthus belliflorus* is currently known from only two populations of less than 100 km^2^ in the northeast of Thailand and the current investigation revealed fewer than 250 mature individuals, it is considered Critically Endangered (CR) according to criteria B1a (i, ii, v), B2a (i, ii, v), and C2a (ii) of the IUCN Red List (IUCN Standards and Petitions Committee, 2022). Nevertheless, there should be further studies to confirm conservation status of this species.

**Additional specimens examined.** THAILAND. Khon Kaen: Wiang Kao District, Phu Wiang National Park, 6 July 2006, *P. Chantaranothai* 23/2006 (KKU!).

## Discussion

The newly discovered taxon, *Nymphanthus belliflorus*, is assigned to *Nymphanthus* section *Nymphanthus* based on its pedicellate flowers, 3-locular ovaries and bifid stigmas. It closely resembles to *N. chantaranothaii*, *N. glaucescens* and *N. huamotensis* in having staminate flowers with four sepals bearing long-fimbriate margins and the pistillate flowers composed of 5–6 sepals with long-fimbriate margins. However, it can be distinguished by its swollen stem base (not swollen in other species), young branchlets that are glabrous (puberulous in other species), disc glands of staminate flowers are obdeltoid and yellow-reddish (reniform and red in other species), pistillate flowers with long pedicel, 3.2–4.8 cm long (1.2–1.4 cm long in *N. chantaranothaii*, 1.7–2.5 cm in *N. glaucescens* and 1.1–1.3 cm in *N. huamotensis*), fruiting pedicel with 3.5–5.5 cm long (1.2–1.5 cm long in *N. chantaranothaii*, 1.5–2.2 cm in *N. glaucescens* and 1.3–1.4 cm in *N. huamotensis*), fruit diameter ranges from 3.3–4.8 mm (2.5–4 mm in *N. chantaranothaii*, ca. 2.5 mm in *N. glaucescens* and 2.5–3 mm in *N. huamotensis*) and seed length ranges 2.3–2.5 mm (1.7–2.0 mm in *N. chantaranothaii*, 2–3 mm in *N. glaucescens* and 1.5–1.6 mm in *N. huamotensis*) (Table 1).

**Key to the species of Thai *Nymphanthus***
  (modified from Flora of Thailand (Chantaranothai, 2007) with additional diagnostic characters incorporated from the present study)
1a. Margin of sepals entire 2
1b. Margin of sepals fimbriate 3
2a. Leaf blades obliquely ovate, 5–12 by 2.5–4 cm ***N. longifolius***
2b. Leaf blades oblong, 0.3–0.5 by 0.05–0.1 cm ***N. taxodiifolius***
3a. Leaves puberulous on upper or both surfaces 4
3b. Leaves glabrous 5
4a. Leaf blade oblong or obovate, 1–2.1 by 0.5–0.8 cm, puberulous on upper surface. Pistillate flowers solitary in leaf-axils along upper half of the branchlets ***N. chantaranothaii***
4b. Leaf blade obliquely ovate, 2.9–3.7 by 1.5–2.1 cm, puberulous on both surfaces. Pistillate flowers solitary in bare racemes in distal axils, sometimes in leaf-axils ***N. tetrandrus***
5a. Branchlets glabrous 6
5b. Branchlets pubescent, puberulous or furfuraceous 8
6a. Leaves more than 5 cm long. Capsules inflated ***N. elegans***
6b. Leaves less than 5 cm long. Capsules not inflated 7
7a. Stem base swollen. Margin of sepals long fimbriate. Pistillate flower pedicels at least 2 cm long ***Nymphanthus belliflorus* P. Sukkharom, Chantar. & Pornp., *sp.nov*.**
7b. Stem base not swollen. Margin of sepals short fimbriate. Pistillate flower pedicels up to 2 cm long ***N. sootepensis***
8a. Ovary glabrous ***N. glaucescens***
8b. Ovary papillose-puberulous or villous 9
9a. Undershrubs to 30 cm high. Leaf blades broadly ovate, obovate, rounded, broadly elliptic or ovate-oblong, venation reddish ***N. huamotensis***
9b. Shrubs to 3 m high. Leaf blades oblong, oblong-lanceolate or lanceolate, venation not reddish 10
10a. Leaf blades lanceolate, 4–7.5 by 2–2.5 cm, petioles 2–3 mm long. Sepals of pistillate flowers 3–5 by 2–3 mm ***N. gracilis***
10b. Leaf blades oblong to oblong-lanceolate, 1.3–2.6 by 0.4–0.9 cm, petioles 0.5–1 mm long. Sepals of pistillate flowers 2.2–3 by 1.2–1.8 mm ***N. pulchroides***

**Table 1:** Comparison of morphological characteristics of *N. belliflorus, N. chantaranothaii*, *N. glaucescens* and *N. huamotensis*.

Characteristics	*N. belliflorus*	*N. chantaranothaii*	*N. glaucescens*	*N. huamotensis*
**Habit**	Undershrub, up to 0.6 m	Shrub, up to 1 m	Shrub, up to 1.2 m	Undershrub, up to 0.3 m
**Stem base**	Swollen	not swollen	not swollen	not swollen
**Young branchlet**	Glabrous	Puberulous	Puberulous	Puberulous
**Leaf**				
Shape	Ovate, obovate, oblong	Oblong, obovate	Ovate, obovate, elliptic	Broadly ovate, rounded
Width	0.2–0.8 cm	0.6–0.8 cm	0.6–1.8 cm	0.2–0.7 cm
Length	0.2–1.7 cm	1–1.8 cm	1–3 cm	10.2–0.8 cm
Texture	Membranous	Membranous	Subcoriaceous	Subcoriaceous
**Staminate flower**				
Sepal	4, red with long white fimbriate margin, rhombic-ovate	4, red with long white fimbriate margin, rhombic-ovate	4–6, red with white long fimbriate, lanceolate	4, red with long fimbriate margin, rhombic-ovate
Disc gland	Yellow with reddish at the lower part, obdeltoid	Red, reniform	Red, reniform	Red, reniform
Arrangement	1–3 flowers in a fascicle, the lower part of the branchlets	2–3 flowers in a fascicle, the lower part of the branchlets	2–6 flowers in a fascicle, the lower part of the branchlets	2–4 flowers in a fascicle, the lower part of the branchlets
Pedicel length	0.6–0.8 cm	0.6–1.2 cm	0.8–1.2 cm	0.7–1 cm
**Pistillate flower**				
Sepal	(5–)6, red with long fimbriate margin, narrowly lanceolate	6, red with long white fimbriate margin, narrowly lanceolate	6, white with red at the lower part and long fimbriate margin, lanceolate	5 or 6, red with long fimbriate, lanceolate
Disc gland	6, annular, yellowish, surface undulate or tuberculate	6, free, yellowish, obovate with truncate apex, surface undulate	5 or 6, annular, yellowish, surface tuberculate	5 or 6, free, red, obovate with truncate apex, surface undulate
Arrangement	Solitary in distal axils	Solitary in the upper part of axils	Solitary in the distal or upper part of axils	Solitary in distal axils
Pedicle length	(2–)3.2–4.8 cm	1.2–1.4 cm	1.7–2.5 cm	1.1–1.3 cm
**Capsule**				
Diameter	3.3–4.8 mm	2.5–4 mm	ca. 2.5 mm	2.5–3 mm
Surface	Smooth	Smooth	Smooth	Papillose
Pedicel length	3.5–5.5 cm	1.2–1.5 cm	1.5–2.2 cm	1.3–1.4 cm
**Seed**				
Width	1.3–1.4 mm	1.0–1.4 mm	0.3–0.5 mm	1.0–1.2 mm
Length	2.3–2.5 mm	1.7–2.0 mm	2–3 mm	1.5–1.6 mm
Length-to-width ratio	1.78:1	1.54:1	6.25:1	1.41:1
Shape	Trigonous	Trigonous	Trigonous	Trigonous
Surface ornamentation	Transversely granular striate	Transversely granular striate	No data	Transversely granular striate

## Conclusions

This study confirmed that *Nymphanthus belliflorus* is a new species that is supported by the distinct morphological characteristics.

## Additional Information and Declarations

### Field Study Permissions

The following information was supplied relating to field study approvals (*i.e*., approving body and any reference numbers):

The reported sample collection location is not in preservation area, and no particular authorizations were required for this area. For additional specimen recorded from preservation area, only herbarium specimens were examined. Our fieldwork did not involve any endangered or protected species. Since the species are currently undescribed, they are not currently included in the Threatened Plants of Thailand (Chamchumroon et al., 2017). No specific permits were required for the present study.

### Data Availability

The following information was supplied regarding data availability:

The comparison of morphological data are available in Table 1

### New Species Registration

The following information was supplied regarding the registration of a newly described species:

*Nymphanthus belliflorus*

Species name: urn:lsid:ipni.org:names:77372199-1
